# Sexual selection on bushcricket genitalia operates in a mosaic pattern

**DOI:** 10.1002/ece3.6025

**Published:** 2020-02-25

**Authors:** Nadja C. Wulff, Gerlind U. C. Lehmann

**Affiliations:** ^1^ Department of Biology, Evolutionary Ecology Humboldt University Berlin Berlin Germany

**Keywords:** copulatory courtship, cryptic female choice, genitalia, mosaic selection, sexual antagonistic coevolution, sexual selection, titillators

## Abstract

In most species with internal fertilization, male genitalia evolve faster than other morphological structures. This holds true for genital titillators, which are used exclusively during mating in several bushcricket subfamilies. Several theories have been proposed for the sexual selection forces driving the evolution of internal genitalia, especially sperm competition, sexually antagonistic coevolution (SAC), and cryptic female choice (CFC). However, it is unclear whether the evolution of genitalia can be described with a single hypothesis or a combination of them. The study of species‐specific genitalia action could contribute to the controversial debate about the underlying selective evolutionary forces.

We studied female mating behaviors in response to experimentally modified titillators in a phylogenetically nested set of four bushcricket species: *Roeseliana roeselii*, *Pholidoptera littoralis littoralis*,* Tettigonia viridissima* (of the subfamily Tettigoniinae), and *Letana inflata* (Phaneropterinae). Bushcricket titillators have several potential functions; they stimulate females and suppress female resistance, ensure proper ampulla or spermatophore attachment, and facilitate male fixation. In *R. roeselii*, titillators stimulate females to accept copulations, supporting sexual selection by CFC. Conversely, titillator modification had no observable effect on the female's behavior in *T. viridissima.* The titillators of *Ph. l. littoralis* mechanically support the mating position and the spermatophore transfer, pointing to sexual selection by SAC. Mixed support was found in *L. inflata*, where manipulation resulted in increased female resistance (evidence for CFC) and mating failures by reduced spermatophore transfer success (evidence for SAC). Sexual selection is highly species‐specific with a mosaic support for either cryptic female choice or sexually antagonistic coevolution or a combination of both in the four species.

## Graphical abstract

Genitalic titillators in male bushcrickets are fast evolving morphological structures. The bushcricket titillators have several functions; they stimulate females and suppress female resistance, ensure proper spermatophore attachment, and facilitate male fixation. Sexual selection seems to act in a mosaic pattern of cryptic female choice and sexual antagonistic coevolution on the genitalia of these species.

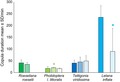

## 1 INTRODUCTION

In most species with internal fertilization, male genitalia evolve much faster than other morphological structures (Eberhard, 1985, 2010a; Rowe & Arnqvist, 2012; Shapiro & Porter, 1989). Evidence is accumulating that the high variability of genitalia can best be explained by mechanisms of sexual selection (Eberhard, 1985, 2009; Hosken & Stockley, 2004; Simmons, 2014; Simmons, House, Hunt, & Garcia‐Gonzalez, 2009). Many—sometimes conflicting—theories have been proposed for the sexual selection forces driving the evolution of internal genitalia (Arnqvist, 1998; Arnqvist & Rowe, 2005; Briceño & Eberhard, 2017; Eberhard, 1985, 1996, 2010a, 2010b, 2011; Hosken & Stockley, 2004; Simmons, 2014). These range from sperm competition (Parker, 1970; Simmons, 2001; Waage, 1979), sexually antagonistic coevolution (Arnqvist & Rowe, 2002; Parker, 1979; Rice, 1996), to cryptic female choice (Eberhard, 1996; Eberhard & Lehmann, 2019; Thornhill, 1983). There is strong evidence for the evolution of insect genitalia under cryptic female choice at least in the broadly studied tsetse flies and a bushcricket species (Eberhard & Lehmann, 2019). However, we also see prime examples of insect genitalia fulfilling the criteria for sexually antagonistic coevolution, especially water striders and beetles of the genus *Callosobruchus* (summarized in Simmons, 2014). Given the range of proposed hypotheses and the cumulating evidence for alternative sexual selection forces in different species, it is unclear whether the evolution of genitalia can be described with a single hypothesis. We still know little about how species‐specific genitalia contribute to the controversial debate about the underlying selective evolutionary forces. Given the species‐specific morphology and the proposed varying function of genitalia, it is possible that criteria supporting different sexual selection theories might be fulfilled in closely related species or even within a single species. Such a mosaic of sexual selection forces acting between and within species might in part explain the long‐standing controversy around genitalia evolution.

Males of several bushcricket subfamilies possess spiny genital organs which are part of the male's phallus (Chamorro‐Rengifo & Lopes‐Andrade, 2014). These sclerotized "titillators" exist in various quantities, structures, and shapes, ranging from simple fields of small tubercles up to a double pair of long and spine‐bearing titillators, depending on the Tettigoniidae subfamily (Lehmann, Gilbert, Vahed, & Lehmann, 2017; Vahed, Lehmann, Gilbert, & Lehmann, 2011). The morphological features of the genital appendages are well described and used for taxonomic purposes (Harz, 1969; Rentz, 1985, 1993, 2001). However, information on the titillators' function in the mating process is still limited to experimental studies on a single species (Wulff, Kamp, Santos Rolo, Baumbach, & Lehmann, 2017; Wulff, Lehmann, Hipsley, & Lehmann, 2015; Wulff & Lehmann, 2014, 2016; Wulff, Schöneich, & Lehmann, 2018) or comparative, nonexperimental, data from a larger number of species (Lehmann et al., 2017; Vahed et al., 2011). These investigations showed that the males' titillators are used during copulation to tap rhythmically on the surface of the female's flap‐like genital fold, which covers the opening of the genital chamber (Wulff et al., 2017, 2015, 2018). In the Tettigoniinae *R. roeselii*, the most studied species of bushcricket in terms of internal genitalia, titillators are not involved in sperm removal (Wulff et al., 2015). However, females can sense stimulation on their genital fold (Wulff et al., 2018) and showed resistance behavior during copulation with males bearing unilaterally shortened titillators (Wulff & Lehmann, 2016; Wulff et al., 2018). Thus, the paired titillators, in this species, act as copulatory courtship devices, both stimulating females by their rhythmic copula movements (Wulff et al., 2017, 2018) and supporting spermatophore transfer (Wulff et al., 2015; Wulff & Lehmann, 2016). Moreover, comparative studies found that males bearing titillators copulated longer than those without (Vahed et al., 2011), and the female's refractory period was shorter in species with more complex titillators (Lehmann et al., 2017). Consequently, the compiled data for the bushcricket *R. roeselii* show that titillators in this species evolved under cryptic female choice (Eberhard & Lehmann, 2019), but sexually antagonistic coevolution might also act in bushcrickets. In the first case, titillators should be used as copulatory courtship devices to stimulate the females, while in the latter case, they could be used for grasping and position securing, allowing males to control the copulation duration, or even wound the females (Dougherty et al., 2017). It has been suggested that genital evolution is influenced simultaneously or sequentially by different sexually selective forces (Eberhard, 2011; Hosken & Stockley, 2004) and that these may have unequal effects on reproductive behavior and genital morphology (Eberhard, 2011). In this paper, we examine whether the species‐specific morphology of the bushcricket titillators can be explained by a unifying function or if sexual selection has led to a variety of functions.

**Figure 1:**
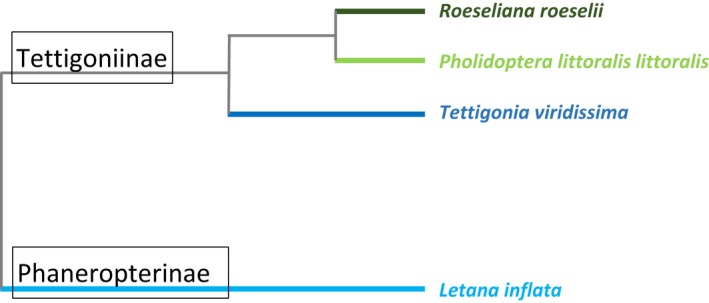
Schematic overview of the nested phylogenetic relationship of the four bushcricket species, combined after Hawlitschek et al. (2017) and Mugleston et al. (2018)

We address this through experiments that alter the titillator structures in three bushcricket species that have stepwise phylogenetic relationships to our model species *Roeseliana roeselii* (Wulff et al., 2017, 2015, 2018; Wulff & Lehmann, 2014, 2016) (Figure 1)*.* Two species were selected from the same subfamily Tettigoniinae, which have paired titillators with numerous spines. A third species was chosen from the different subfamily Phaneropterinae, bearing a single titillator.

**Figure 2:**
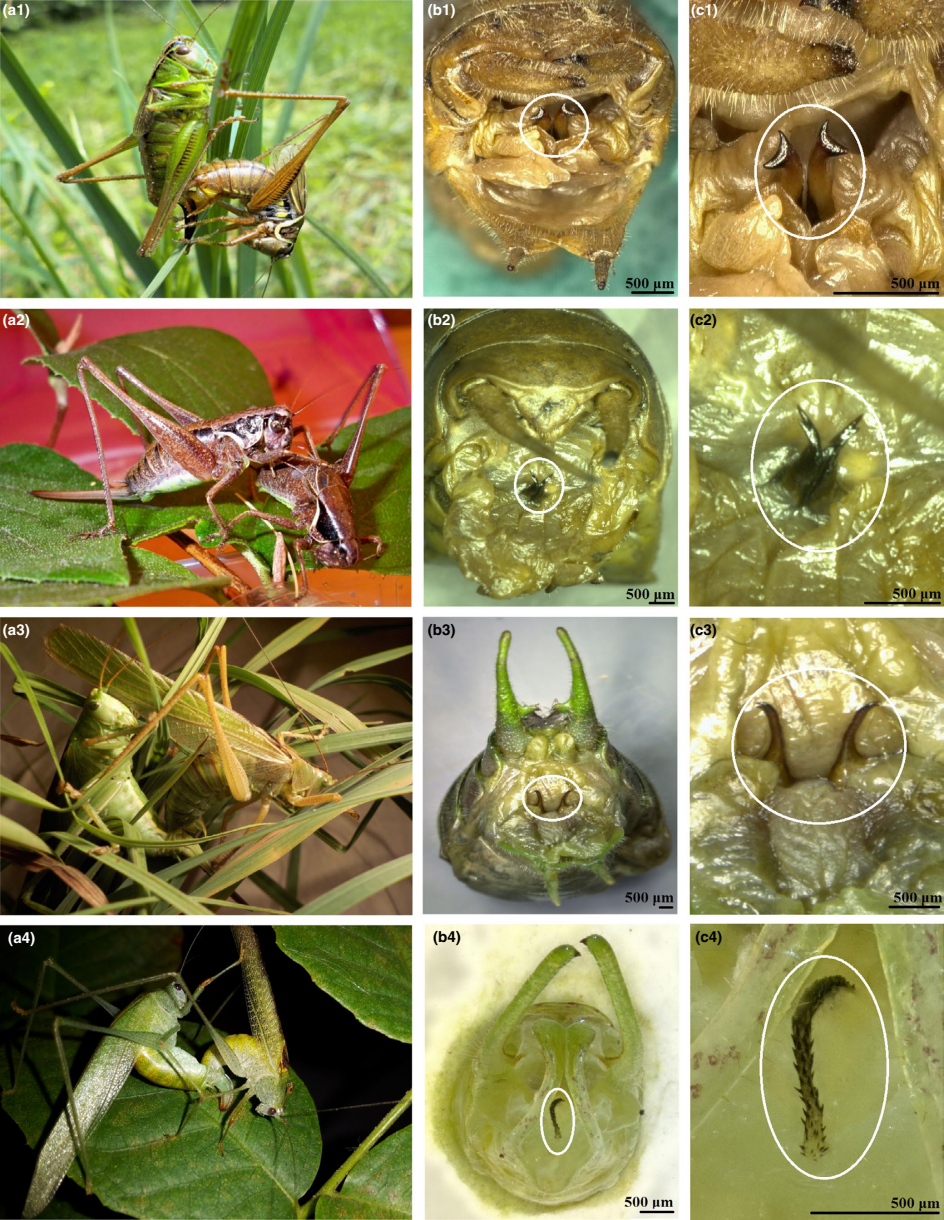
Mating pairs with females to the left (left column: 1a–4a), the male titillators encircled in white (middle column: 1b–4b) and enlarged (right column: 1c–4c) of the four species (1a–c) *Roeseliana roeselii*, (2a‐c) *Pholidoptera l. littoralis*, (3a–c) *Tettigonia viridissima* and (4a–c) *Letana inflata*. Scale bars for column b and c show 500 µm

Mating in bushcrickets can be described along behavioral landmarks (compare Lehmann & Lehmann, 2008; Wulff & Lehmann, 2016); once a male and a female have physical contact with their antennae, the male tries to achieve the mating position. Copula is initiated by grasping the female with a male's cerci, sometimes supported by the subgenital plate holding her ovipositor. Once a firm coupling is established, the female opens her subgenital plate to give the male access to her genital chamber. The male pulls near the female to make close contact and insert his titillators into the female' genital chamber. The titillator together with the male's phallobasis is then rhythmically moved forwards and backwards. Two types of titillator movements can be distinguished. During the small ones, the titillator is moved inside the female, whereas in the big ones, the titillator is moved in and out, becoming visible during retraction phases. Both types of titillator movements can be observed without manipulation (Video S1).

In the three Tettigoniinae species, the males transfer a large spermatophore at the end of the mating, containing a spermatophylax and the ampulla with the male's sperm (Lehmann et al., 2018; Vahed et al., 2011). While the female eats the spermatophylax, the sperm migrates from the ampulla into the female's seminal receptacle (Lehmann, 2012). In the subfamily Phaneropterinae, the majority of the roughly 3,000 species (Cigliano, Braun, Eades, & Otte, 2019) have no titillators. One notable exception is the species *Letana inflata.* Males have one spiny titillator and transformed genital lobes, which they use as claspers to restrain the female after the transfer of the sperm‐containing ampulla (Heller & Liu, 2015). The prolonged mate guarding in this species prevents the females from eating the ampulla and gives the sperm the time it needs to migrate successfully into the female's body (Lehmann, Heller, & Mai, 2016).

To test for selective forces likely to explain the evolution of titillators, we observed the responses of females mated to males of the wild type or with experimentally altered genital titillators. If they are sexually selected, we hypothesize that titillator manipulations affect female behavior during or after copula. Based on the main hypotheses for sexual selection on genitalia, cryptic female choice, and sexually antagonistic coevolution, we developed a matrix for likely copulatory and postcopulatory responses (see Table 5), largely orienting on the extensive list for separating CFC from alternatives, given in Eberhard and Lehmann (2019). As we tested both symmetric and asymmetric titillator‐manipulated males, we expanded the predictions to the symmetry type. Cryptic female choice postulates that male genitalia function to stimulate the female; hence, a female receives information about a male's quality by his copulatory courtship. A titillator‐manipulated male might therefore show a reduced speed of titillator movements. This altered copulatory courtship and the sensed alteration of titillator form might lead to a struggling behavior of the female during copulation. The lack of any mechanical fixation or manipulation by the male clearly distinguishes cryptic female choice from sexually antagonistic coevolution, where titillators might mechanically facilitate male physical attachment. Manipulative ablation of titillators might increase the number of mating failures, while the reduced mechanical restrictions might allow males to increase their movement speed. Moreover, the spermatophore transfer efficiency could also be affected.

A similar response to titillator manipulation in all four species would support a single sexual selection force responsible for the evolution of titillators. In contrast, mating responses differing between species would provide support for a mosaic of forces acting, especially when there is evidence for cryptic female choice and sexually antagonistic coevolution within a species.

## 2 MATERIALS AND METHODS

### 2.1 Study species

Four bushcricket species were used three European Tettigoniinae (a) *R. roeselii* (Hagenbach, 1822) previously known under *Metrioptera roeselii* (see Wulff et al., 2015, 2017, 2018; Wulff & Lehmann, 2014, 2016), (b) *Pholidoptera littoralis littoralis* (Fieber, 1853), (c) *Tettigonia viridissima* (Linnaeus, 1758), and (d) one Asian tropical bushcricket of the subfamily Phaneropterinae, *L. inflata* (Brunner von Wattenwyl, 1878) (Figure 2). The species are selected along a phylogenetic gradient with the nested order [{(*R. roeselii* – *Ph. l. littoralis*) – *T. viridissima*} – *L. inflata*] (Hawlitschek et al., 2017; Mugleston, Naegle, Song, & Whiting, 2018) (Figure 1).

The males of the three Tettigoniinae species bear paired titillators with several spines on the tips (Harz, 1969; Lehmann et al., 2017; Vahed et al., 2011), whereas *L. inflata* males possess a single titillator with several spines, which is merged with the surrounding tissue of the phallobasis (Heller & Liu, 2015) (Figure 2).

Individuals of the three tettigoniids were caught as juveniles in the wild and reared to adulthood in the laboratory (Table 1). The individuals of *L. inflata* originated from a single female captured in Sri Lanka (Heller & Liu, 2015). Animals were reared until adulthood in groups of about 7–20 individuals per container (dimensions: height 40 cm × width 60 cm × depth 40 cm), depending on the animal size. Before reaching sexual maturity, adults were separated and individually accommodated in 0.5‐L plastic containers covered with gauze. All individuals were fed their species‐specific diet ad libitum (Table 1), and water was sprinkled once to twice a day on the walls of the boxes and plastic jars. Ambient temperature in the laboratory was 22–25°C with a light–dark cycle of 16:8 hr.

**Table 1:** Collection sites and feeding regimes of the four bushcricket species

Species name	Collected from	Date	Feeding regime ad libitum
*Roeseliana roeselii*	Germany, two localities in and around Berlin 52°25′41″N, 13°11′56″E 52°23′14″N, 13°12′54″E	2015	Fresh grass Oat flakes Bee pollen Dried fish food pellets (Tetramin^®^) Crickets and bushcrickets: dead (for *R. roeselii* and *Ph. l. littoralis*) alive (for *T. viridissima*)
*Tettigonia viridissima*		2016	
*Pholidoptera l. littoralis*	Slovenia, Gabrče 45°42′47″N, 14°01′22″E	2015	
*Letana inflata*	Sri Lanka, Ella 8°52′N, 81°3′E 500 m a.s.l.	2014	Leaves of *Lactuca sativa* and *Taraxacum officinale*

### 2.2 Titillator manipulations

To test for changes in female mating behaviors as a response to manipulations, the male's titillator(s) were shortened or covered with UV‐hardening glue before mating experiments (Table 2). The males of each species were assigned randomly to one of the treatment groups (i.e., manipulation or sham operation). The paired titillators of the three Tettigoniinae species were shortened with fine scissors (No. 15024‐10, Fine Science Tools GmbH, Heidelberg) under a stereo microscope (Wild M5A, Wild Heerbrugg AG). The effects of the ablation of one or two titillators in *R. roeselii* have already been described (Wulff & Lehmann, 2016). In the current study, just the spines on the tips of the left titillator were ablated (_p_T_‐left spines_), to test the effects of titillator asymmetry found previously for the removal of the whole tip, bearing the spines (Wulff & Lehmann, 2016; Wulff et al., 2018). For *T. viridissima* and *Ph. l. littoralis*, the outer parts of the left paired titillator (_p_T_‐1_) or of both titillators (_p_T_‐2_) were removed. The wild‐type males were handled identically with the titillators touched with the cutting edges of fine scissors but leaving the titillators intact. The single titillator in *L. inflata* is merged with the surrounding tissue (Heller & Liu, 2015, see Figure 1). As removal of the titillator was therefore not an option, we covered the spines on the single titillator (_s_T_glued_) with UV‐hardening glue (UV‐Star, Marston‐Domsel GmbH). The glue was applied precisely on the spines with the tip of a fine long brush‐hair under the stereo microscope and hardened for 30 s with a UV‐Lamp (“UV‐Beamer,” Marston‐Domsel GmbH). In the wild‐type group, the single titillator was touched with the tip of the fine brush‐hair, and, to control for possible side effects of the glue on the males without interfering with the copulation, it was applied on the basal part of the male's genital lobe. After application and hardening of the UV‐glue, its correct and firm placement was verified.

**Table 2:** Manipulation scheme for the four bushcricket species. Wild type: sham operation; _p_T_‐2_: both titillators ablated; _p_T_‐1_: the left titillator ablated; _p_T_‐left spines_: spines on the tips of the left titillator removed; _s_T_glued_: spines of the single titillator covered with UV‐hardening glue

Species	Number of titillator(s)	Wild types (sham operated)	_p_T_‐1_ (one titillator ablated)	_p_T_‐2_ (both titillators ablated)	Other manipulations
*Roeseliana roeselii*	2‐Paired (_p_T)	*n* = 20	[Previous experiments: see Wulff & Lehmann, 2016]	[Previous experiments: see Wulff & Lehmann, 2016]	_p_T_‐left spines_ *n* = 21
*Pholidoptera l. littoralis*	2‐Paired (_p_T)	*n* = 13	*n* = 6	*n* = 23	
*Tettigonia viridissima*	2‐Paired (_p_T)	*n* = 21	*n* = 15	*n* = 21	
*Letana inflata*	1‐Single (_s_T)	*n* = 16			_s_T_glued_ *n* = 18

### 2.3 Mating experiments

The mating partners were mated in a dome‐shaped meshed arena (30 × 30 × 20 cm), allowing the pairs to hold tight to the meshes and achieve mating position. Males of the three species *R. roeselii*,* Ph. l. littoralis*, and *T. viridissima*, bearing paired titillators, were allowed to recover from potential handling stress for one day before mating. Individuals of the single titillator possessing *L. inflata* were used immediately after UV hardening, because some individuals were able to remove the glue from their genitalia over time. In line with their natural activity time, *R. roeselii* was tested during the daytime, whereas *T. viridissima*, *Ph. littoralis*, and *L. inflata* were mated at night between 10 p.m. and 6 a.m. Prior to the experiments, all males and females were weighed on a precision balance (Kern EG 300 –3 M, 0.001/300 g). Randomization of males and females was successful regarding body mass of three species, only in *T. viridissima* were males of one out of three groups significantly lighter (Table 3).

**Table 3:** Body masses (mean ± *SD*) of the Tettigoniinae *Roeseliana roeselii*, *Pholidoptera l. littoralis*,* Tettigonia viridissima*, and the Phaneropterinae species *Letana inflata* separated for sex and treatment groups

Species	Sex	Wild types	_p_T_‐1_	_p_T_‐2_	Other manipulations	Statistics
		(sham operated)	(one titillator ablated)	(both titillators ablated)		
*Roeseliana roeselii*	*Males*	297.15 ± 35.41 (*n* = 20)			290.81 ± 39.17 (*n* = 21)	*t* Test: *t*_39_ = 0.54, *p* = .59
	*Females*	498.30 ± 67.07 (*n* = 20)			483.81 ± 68.59 (*n* = 21)	*t* Test: *t*_39_ = 0.68, *p* = .50
*Pholidoptera littoralis littoralis*	*Males*	1,379.38 ± 60.59 (*n* = 13)	1,371.50 ± 84.07 (*n* = 6)	1,368.09 ± 90.68 (*n* = 23)		ANOVA: *F*_2,39_ = 0.080, *p* = .92
	*Females*	1,820.98 ± 180.86 (*n* = 13)	1,798.83 ± 113.22 (*n* = 6)	1,782.48 ± 168.84 (*n* = 23)		ANOVA: *F*_2,39_ = 0.21, *p* = .81
*Tettigonia viridissima*	*Males*	1,329.26 ± 164.66 (*n* = 19)	1,240.13 ± 153.24 (*n* = 15)	1,322.05 ± 159.40 (*n* = 20)		ANOVA: *F*_2,51_ = 1.56, *p* = .22
	*Females*	2,366.50 ± 297.22 (*n* = 20)	2,402.13 ± 221.10 (*n* = 15)	2,348.21 ± 295.61 (*n* = 19)		ANOVA: *F*_2,51_ = 0.16, *p* = .85
*Letana inflata*	*Males*	172.17 ± 28.56 (*n* = 6)			181.33 ± 12.24 (*n* = 6)	*t* Test: *t*_10_ = −0.72, *p* = .49
	*Females*	423.40 ± 82.12 (*n* = 10)			463.88 ± 50.57 (*n* = 8)	*t* Test: *t*_16_ = −1.22, *p* = .24

Ten mating‐related parameters were measured or observed in real time following previously established protocols (Wulff & Lehmann, 2016). Six parameters (1–6) plus two subparameters (1a,1b) are linked to copulation, the other four (7–10) measured postcopulatory outcomes (Table 4).

**Table 4:** Mating‐related parameters of six copulatory and four postcopulatory characters measured or observed in the four bushcricket species *Roeseliana roeselii*, *Pholidoptera l. littoralis*, *Tettigonia viridissima*, and *Letana inflata*

		Trait	Unit	*Roeseliana roeselii*	*Pholidoptera l. littoralis*	*Tettigonia viridissima*	*Letana inflata*
Copulatory	1	Copula duration^a^	min		–b		
	1a	Separations during copula^c^	*n*=			**Does not occur** d	
	1b	Uninterrupted last part of copula^e^	min				
	2	Titillator movements big^f^	*n*=/min				**No data** g
		Titillator movements small^h^	*n*=/min				
	3	Female mating resistance^i^	%				
	4	Failed titillator anchoring^j^	%				
	5	Spermatophore transfer success^k^	%	–l	–l	–l	–m
	6	Spermatophore transfer duration	sec				
Postcopulatory	7	Spermatophore mass^n^	mg				**No data** o
	8	Spermatophore consumption duration	min	–p	**No data** q	–p	–r
	9	Refractory period s	days			**No data**	
	10	Egg number^t^	*n*=		**No data**	**No data**	

Explanations for traits with lacking data are marked in bold.

a Total time from coupling the male cerci to the female until separation of the pair after spermatophore transfer.

b As couples repeatedly separate during copula, all single copula events were summed up.

c Defined as the number a pair interrupts the cerci coupling and reengage in copula.

d Does not occur in *T. viridissima*.

e Duration of the last copula attempt, leading to the spermatophore transfer or the termination of mating.

f Visible retraction of parts or the total male titillators out of the female and reinsertion (in‐and‐out movement).

g Titillator movements are not external visible in *L. inflata* (Lehmann et al., 2017).

h Visible movement of the male titillators inside the female without retraction.

i Occurrence of female walking, jumping, kicking, and eventually biting during copulation. Percentage of females showing this behavior.

j Failed mechanical anchoring of the titillator, leading to slipping out of the copula position with a full retraction of the titillators.

k Percentage of mating couples successfully ending their mating by transfer of a spermatophore.

l The bushcricket spermatophore consists of the sperm‐bearing ampulla and a gelatinous nutritious spermatophylax.

m However, in *L. inflata* the spermatophore is deposited inside the female genital chamber and is built only of the ampulla.

n Spermatophores were removed after copulation using fine forceps and immediately weighed on a precision balance (Kern EG 300 –3 M, 0.001/300 g).

o Transfers an internal ampulla that is not accessible without dissection (Lehmann et al., 2017).

p Spermatophore consisting of the sperm‐containing ampulla and a surrounding gelatinous spermatophylax.

q No precise data taken—the females took several hours to finish ingestion.

r Spermatophore build only by the sperm‐containing ampulla.

s Females were presented every day a virgin male ready‐to‐mate to test for female willingness to remate.

t Number of eggs laid until remating.

To test for the different hypotheses of sexual selection acting on bushcricket titillators, we have developed specific predictions for the six copulatory and four postcopulatory traits (Table 5). Many of the predictions can be deduced from our list supporting cryptic female choice in insect genitalia of tsetse flies and the bushcricket *R. roeselii* (Eberhard & Lehmann, 2019). Cryptic female choice and sexually antagonistic coevolution make distinct predictions for the outcomes in mating with genitalia‐manipulated males. As we have seen different responses between symmetrically and asymmetrically manipulated titillators in *R. roeselii* (Wulff & Lehmann, 2016; Wulff et al., 2018), we extended the predictions for the number of titillator movements regarding symmetry.

**Table 5:** Relevance of the mating‐related parameters during and after copulation for sexual selection, especially to distinguish between cryptic female choice (CFC) and sexually antagonistic coevolution (SAC)

	Trait	Implications for sexual selection theory	
		Predicted results under CFC (cryptic female choice)	Predicted results under SAC (sexually antagonistic coevolution)
Copulatory			
1 & 1b^a^	Copula duration and uninterrupted last part of copula	Prolonged: Males need more time to properly stimulate the female or Shortened: Males are less able to stimulate females to get longer copulations accepted	Prolonged: Males need more attempts to manipulate the females adequately or Shortened: Males will fail or be less able to manipulate females into longer copula
1a^a^	Number of separations during copula	Unaltered: no sense to reengage with an inferior stimulating male	Increased: Males are less able to enforce a longer copulation
2	Titillator movement number	Increased: Males try to compensate the reduced stimulatory capacity of the altered titillators (symmetric > asymmetric) or Decreased: If females react toward the less stimulatory effect of altered titillators, males might reduce this investment (symmetric > asymmetric)	Increased: Males need less mechanical force, therefore can accelerate titillator movements (symmetric > asymmetric)
3^a^	Female mating resistance	Increased: Males stimulate females less successful, not able to distract her from resistance	Unaltered
4	Failed mechanical anchoring of the titillator	Unaltered: The titillator functions to stimulate not as an anchor	Increased: Males are less able to anchor their titillators
5	Success of spermatophore transfer	Unaltered	Reduced: Failures to mechanically support the spermatophore transfer
6^a^	Duration of spermatophore transfer	Decreased: Females might choose to terminate copulations	Decreased: Males might be less able to coercively prolong copulations
Post‐copulatory			
7	Spermatophore mass	Unaltered: It is under male control and might be unaffected by female responses	
8	Consumption duration of the spermatophore	Unaltered: It correlates strongly with spermatophore mass, see trait seven	
9	Refractory period until female remating	Unaltered: It correlates strongly with the amount of transferred ejaculate and lesser with the amount of consumed spermatophylax, which both correlate with spermatophore attachment duration, see trait eight	
10	Egg number until remating	Unaltered: It correlates strongly with the refractory period, see trait nine	

a Changes indicate that titillators are under sexual selection by female choice.

Statistical analysis was performed using Excel and SPSS version 24 (IBM SPSS Statistics 24).

## 3 RESULTS

Female responses during copulations toward titillator‐manipulated males were highly species‐specific (Tables 6 and 7). No evidence for sexual selection on titillators was found in *T. viridissima*, as the removal of one or both titillators had no effect on the mating outcome, nor female or male mating behaviors. However, the altered female behaviors in the other three species showed no consistent pattern as responses were not correlated with the morphology of the titillators, asymmetric or symmetric alterations, nor phylogenetic relationships (see Tables 6 and aggregated summary in Table 7).

**Table 6:** Data from six copulatory and four postcopulatory characters in the Tettigoniinae *Roeseliana roeselii*, *Pholidoptera l. littoralis*, and *Tettigonia viridissima* and the Phaneropterinae species *Letana inflata* separated for treatment groups

*Roeseliana roeselii*							
Trait		Unit	Wild type	_p_T_‐2_	_p_T_‐1_	_p_T_‐left spines_	Statistic (wild type vs. _p_T_‐left spines_)
Copulatory							
1	Copula duration^a^	min	41.16 ± 12.87 (*n* = 20) *30.45 ± 7.83 (n = 29)* g	*34.82 ± 9.94 (n = 23)* g	*34.01 ± 7.53 (n = 24)* g	34.82 ± 8.12 (*n* = 21)	*t* Test: *t*_39_ = 1.89, *p* = .066 *ANOVA: Dunnett's post hoc test *versus* wild type: p > .05*
1a	Separations during copula^b^	*n*=	0 (*n* = 20)	*No data*	*No data*	0.33 ± 0.73 (*n* = 21)	Mann–Whitney *U* test: *z* = 1.03, *p* = .30
1b	Uninterrupted last part of copula^b^	min	41.16 ± 12.87 (*n* = 20)	*No data*	*No data*	33.11 ± 8.38 (*n* = 18)	***t* Test: *t*_36_ = 2.25, *p* = .030**
2	Titillator movements: big	*n*=/min	9.93 ± 2.00 (*n* = 20) *10.67 ± 2.47 (n = 29)* g	*9.19 ± 2.84 (n = 24)* g	*9.46 ± 3.19 (n = 24)* g	10.00 ± 2.75 (*n* = 19)	*t* Test: *t*_37_ = 0.087, *p* = .93 **ANOVA: Dunnett's post hoc test wild type versus _p_T**_‐_ **_2_: *p* < .05**
	Small	*n*=/min	18.92 ± 4.65 (*n* = 17)	*No data*	*No data*	16.52 ± 3.93 (*n* = 18)	*t* Test: *t*_33_ = 1.65, *p* = .11
3	Female mating resistance^d^	%^e^	0% (*n* = 20) *4.2% (n = 24)* g	*16.7% (n = 24)* g	*37.5% (n = 24)* g	33.33% (*n* = 21)	**Fisher's exact test: *p* = .0086, *n* = 41** ***Pearson chi‐square: χ2_4_*_,_*_114_ = 12.21*,* p < .001; Dunnett's post hoc comparison wild type *versus* pT−1: χ21*,*46 = 8.08*,* p < .05***
4	Failed titillator anchoring/slipping out	%	Does not apply	Does not apply	Does not apply	Does not apply	
5	Success of spermatophore/ampulla transfer^f^	%	100% (*n* = 20) *100% (n = 28)* g	*70.8% (n = 24)* g	*87.5% (n = 24)* g	85.7% (*n* = 21)	Fisher's exact test: *p* = .23, *n* = 41 ***Pearson chi‐square: χ2_4_*_,_*_111_ = 19.24*,* p < .001*** *;* ***Dunnett's post hoc comparison wild type *versus* pT−2: adjusted χ2_1_*_,_*_48_ = 8.28*,* p < .05***
6	Duration of spermatophore/ampulla transfer^f^	sec	79.75 ± 21.22 (*n* = 20) *82.80 ± 13.20 (n = 28)* g	*88.80 ± 25.80 (n = 22)* g	*85.80 ± 16.20 (n = 24)* g	73.67 ± 21.46 (*n* = 18)	*t* Test unequal variances: *t*_33_ = 1.96, *p* = .058 *ANOVA: Dunnett's post hoc test *versus* wild type: p > .05*
Postcopulatory							
7	Spermatophore^f^	mg	28.50 ± 9.26 (*n* = 20)	*No data*	*No data*	32.24 ± 7.06 (*n* = 19)	*t* Test: *t*_37_=−1.41, *p* = .17
8	Consumption duration of the nuptial gift (spermatophore or ampulla)	min	161.98 ± 79.00 (*n* = 19)	*No data*	*No data*	182.09 ± 83.13 (*n* = 18)	*t* Test: *t*_35_ = 0.75, *p* = .46
9	Female refractory period until remating	days	11.63 ± 10.85 (*n* = 8)	*No data*	*No data*	9.38 ± 9.11 (*n* = 10)	*t* Test: *t*_16_ = 0.48, *p* = .64
10	Egg number until remating	*n*=	9.25 ± 11.20 (*n* = 8)	*No data*	*No data*	4.50 ± 7.59 (*n* = 10)	*t* Test: *t*_16_ = 1.07, *p* = .30

*Pholidoptera l. littoralis*						
Trait		Unit	Wild type	_p_T_‐2_	_p_T_‐1_	Statistic
Copulatory						
1	Copula duration^a^	min	17.41 ± 8.17 (*n* = 13)	16.85 ± 2.87 (*n* = 8)	20.78 ± 0.81 (*n* = 4)	**Kruskal–Wallis test: *χ*^2^_2,22_ = 6.79, *p* = .034**; **Dunn–Bonferroni test: wild type versus _p_T**_‐_ **_1_: *z*** = **−2.59, *p* = .029**; _p_T_‐1_ versus _p_T_‐2_: *z* = 2.05, *p* > .05; wild type versus _p_T_‐2_: *z* = −0.50, *p* > .05
1a	Separations during copula^b^	*n*=	1.00 ± 1.68 (*n* = 13)	2.00 ± 2.66 (*n* = 23)	3.17 ± 1.72 (*n* = 6)	**Kruskal–Wallis test: *χ*^2^_2,24_ = 8.28, *p* = .016**; **Dunn–Bonferroni test: wild type versus _p_T**_‐_ **_1_: *z* = −16.50, *p* = .013**; _p_T_‐2_ versus _p_T_‐1_: *z* = 9.85, *p* = .20; wild type versus _p_T_‐2_: *z* = −6.65, *p* = .31
1b	Uninterrupted last part of copula^b^	min	14.80 ± 9.42 (*n* = 13)	16.18 ± 3.14 (*n* = 8)	8.45 ± 0.77 (*n* = 4)	**Kruskal–Wallis test: *χ*^2^_2,22_ = 10.03, *p* = .007**; Dunn–Bonferroni test: wild type versus _p_T_‐1_: *z* = 9.02, *p* = .096; **_p_T**_‐_ **_1_ versus _p_T**_‐_ **_2_: *z* = −14.25, *p* = .005**; wild type versus _p_T_‐2_: *z* = −5.23, *p* = .34
2	Titillator movements: big	*n*=/min	22.32 ± 3.92 (*n* = 12)	18.73 ± 2.99 (*n* = 8)	24.00 ± 4.00 (*n* = 3)	ANOVA: *F*_2,20_ = 3.30, *p* = .058
	Titillator movements: small	*n*=/min	0	0	0	
3	Female mating resistance^d^	%^e^	0% (*n* = 13)	4.4% (*n* = 23)	16.7% (*n* = 6)	Fisher's exact test: *χ* ^2^_2,42_ = 2.53, *p* = .28
4	Failed titillator anchoring/slipping out	%	38.5% (*n* = 13)	78.3% (*n* = 23)	100% (*n* = 6)	**Fisher's exact test: *χ*^2^_2,42_ = 8.41, *p* = .011**; **wild type versus _p_T**_‐_ **_1_: *χ*2 _1,19_ = 6.38, *p* = .018**; _p_T_‐1_ versus _p_T_‐2_: *p* = .55, *n* = 29; **wild type versus _p_T**_‐_ **_2_: *χ*2_1,36_ = 5.70, *p* = .030**
5	Success of spermatophore/ampulla transfer^f^	%	100% (*n* = 13)	30.4% (*n* = 23)	66.7% (*n* = 6)	**Fisher's exact test: *χ*^2^_2,42_ = 18.33, *p* < .001**; wild type versus _p_T_‐1_: *χ*2_1,19_ = 4.84, *p* = .088; _p_T_‐1_ versus _p_T_‐2_: *p* = .164, *n* = 29¸ **wild type versus _p_T**_‐_ **_2_: *χ*^2^_1,36_ = 16.28, *p* < .001**
6	Duration of spermatophore/ampulla transfer^f^	sec	163.85 ± 27.14 (*n* = 13)	145.75 ± 18.66 (*n* = 4)	144.38 ± 13.84 (*n* = 8)	ANOVA: *F*_2,22_ = 2.22, *p* = .13
Postcopulatory						
7	Spermatophore^f^	mg	158.50 ± 18.66 (*n* = 12)	169.88 ± 15.50 (*n* = 8)	178.63 ± 11.78 (*n* = 4)	ANOVA: *F*_2,21_ = 2.53, *p* = .10
8	Consumption duration of the nuptial gift (spermatophore or ampulla)	min	No data	No data	No data	
9	Female refractory period until remating	days	3.67 ± 2.35 (*n* = 9)	3.50 ± 1.60 (*n* = 8)	2.50 ± 1.73 (*n* = 4)	*F* test: *F*_2,18_ = 0.50, *p* = .61
10	Egg number until remating	*n*=	No data	No data	No data	

*Tettigonia viridissima*						
Trait		Unit	Wild type	_p_T_‐2_	_p_T_‐1_	Statistic
Copulatory						
1	Copula duration^a^	min	40.39 ± 12.56 (*n* = 20)	49.24 ± 22.25 (*n* = 15)	45.25 ± 20.43 (*n* = 21)	ANOVA: *F*_2,53_ = 0.99, *p* = .38
1a	Separations during copula^b^	*n*=	0% (*n* = 21)	0% (*n* = 15)	0% (*n* = 21)	
1b	Uninterrupted last part of copula^b^	min	Does not apply	Does not apply	Does not apply	
2	Titillator movements: big	*n*=/min	4.97 ± 1.60 (*n* = 20)	4.50 ± 0.83 (*n* = 14)	4.87 ± 1.23 (*n* = 21)	ANOVA: *F*_2,52_ = 0.56, *p* = .58
	Titillator movements: small	*n*=/min	37.05 ± 6.12 (*n* = 21)	34.82 ± 4.90 (*n* = 14)	35.29 ± 4.23 (*n* = 21)	ANOVA: *F*_2,53_ = 0.96, *p* = .39
3	Female mating resistance^d^	%^e^	14.3% (*n* = 21)	0% (*n* = 15)	9.5% (*n* = 21)	Fisher's exact test: *p* = .35, *n* = 57
4	Failed titillator anchoring/slipping out	%	Does not apply	Does not apply	Does not apply	
5	Success of spermatophore/ampulla transfer^f^	%	90.5% (*n* = 21)	100% (*n* = 15)	95.2% (*n* = 21)	Fisher's exact test: *p* = .77, *n* = 57
6	Duration of spermatophore/ampulla transfer^f^	sec	325.47 ± 66.06 (*n* = 19)	359.40 ± 49.53 (*n* = 15)	350.05 ± 47.36 (*n* = 20)	ANOVA: *F*_2,51_ = 1.77, *p* = .18
Postcopulatory						
7	Spermatophore^f^	mg	262.95 ± 56.27 (*n* = 19)	226.33 ± 43.51 (*n* = 15)	236.17 ± 34.33 (*n* = 18)	ANOVA: *F*_2,49_ = 2.97, *p* = .060
8	Consumption duration of the nuptial gift (spermatophore or ampulla)	min	835.50 ± 271.34 (*n* = 4)	817.40 ± 263.96 (*n* = 5)	713.44 ± 192.48 (*n* = 9)	ANOVA: *F*_2,15_ = 0.54, *p* = .59
9	Female refractory period until remating	days	No data	No data	No data	
10	Egg number until remating	*n*=	No data	No data	No data	

*Letana inflata*					
Trait		Unit	Wild type	_s_T_glued_	Statistic
Copulatory					
1	Copula duration^a^	min	234.90 ± 49.24 (*n* = 16)	89.93 ± 97.72 (*n* = 18)	***t* Test: t_welch(25.75)_ = 5.55, *p* < .001**
1a	Separations during copula^b^	*n*=	0% (*n* = 16)	11% (*n* = 18)	Fisher's exact test: *p* = .49
1b	Uninterrupted last part of copula^b^	min	Does not apply	Does not apply	
2	Titillator movements: big	*n*=/min	No data^c^	No data^c^	
	Titillator movements: small	*n*=/min	No data^c^	No data^c^	
3	Female mating resistance^d^	%^e^	25.0% (*n* = 16)	72.2% (*n* = 18)	**Pearson** chi‐square **test: *χ*^2^_1,34_ = 7.56, *p* = .0060**
4	Failed titillator anchoring/slipping out	%	Does not apply	Does not apply	
5	Success of spermatophore/ampulla transfer^f^	%	87.5% (*n* = 16)	33.3% (*n* = 18)	**Pearson** chi‐square **test: *χ*^2^_1,34_ = 10.26, *p* = .0014**
6	Duration of spermatophore/ampulla transfer^f^	sec	110.00 ± 31.02 (*n* = 7)	74.00 ± 25.25 (*n* = 8)	***t* Test: *t*_13_ = 2.48, *p* = .028**
Postcopulatory					
7	Spermatophore^f^	mg	No data	No data	
8	Consumption duration of the nuptial gift (spermatophore or ampulla)	min	54.14 ± 51.09 (*n* = 7)	36.83 ± 20.04 (*n* = 3)	Mann–Whitney *U* test: *U* = 8.00, *p* = .67
9	Female refractory period until remating	days	8.40 ± 6.91 (*n* = 5)	7.20 ± 9.63 (*n* = 5)	Mann–Whitney *U* test: *U* = 9.50, *p* = .59
10	Egg number until remating	*n*=	15.60 ± 17.34 (*n* = 5)	14.00 ± 24.87 (*n* = 5)	Mann–Whitney *U* test: *U* = 11.00, *p* = .83

Explanations for traits with lacking data are marked in bold.

a As couples of *Ph. l. littoralis* repeatedly separate during copula, all single copula events were summed up.

b Occurs in *Ph. l. littoralis*,* R. roeselii*, and in *L. inflata* but not *T. viridissima*.

c Titillator movements are not external visible in *L. inflata* (Lehmann et al., 2017).

d Occurrence of female walking, jumping, kicking, and eventually biting.

e Percentage of females showing this behavior.

f Spermatophore (spermatophylax + ampulla).

g Data from previous experiments: Wulff & Lehmann, 2016 (italics).

**Table 7:** Changes in six mating‐related traits as a response to male titillator manipulations for the Tettigoniinae *Roeseliana roeselii*, *Pholidoptera l. littoralis*, *Tettigonia viridissima*, and the Phaneropterinae species *Letana inflata*

	Measured effect	*Roeseliana roeselii*			*Pholidoptera l. littoralis*		*Tettigonia viridissima*		*Letana inflata*
		_p_T_‐2_	_p_T_‐1_	_p_T_‐left spines_	_p_T_‐2_	_p_T_‐1_	_p_T_‐2_	_p_T_‐1_	_s_T_glued_
Copulatory									
1	Copula duration	=	=	=	=	**CFC/SAC**	=	=	**CFC/SAC**
1a	Number of separations during copula	No data	No data	=	=	**SAC**	=	=	=
1b	Uninterrupted last part of copula	No data	No data	**CFC/SAC**	=	=	=	=	Does not apply
2	Titillator movement number	**CFC**	=	=	=	=	=	=	**Not visible**
3	Female mating resistance	=	**CFC/SAC**	**CFC/SAC**	=	=	=	=	**CFC/SAC**
4	Titillator anchoring success	Does not apply	Does not apply	Does not apply	**SAC**	**SAC**	Does not apply	Does not apply	Does not apply
5	Spermatophore transfer success	**CFC/SAC**	=	=	**CFC/SAC**	=	=	=	**CFC/SAC**
6	Spermatophore transfer duration	=	=	=	=	=	=	=	**CFC/SAC**
Postcopulatory									
7	Spermatophore mass	No data	No data	=	=	=	=	=	No data
8	Spermatophore consumption duration	No data	No data	=	No data	No data	=	=	=
9	Female refractory period	No data	No data	=	=	=	No data	No data	=
10	Egg number until remating	No data	No data	=	No data	No data	No data	No data	=
		Previous experiments: see Wulff & Lehmann, 2016	Previous experiments: see Wulff & Lehmann, 2016, Wulff et al., 2018						

Note

Directional changes toward longer copula duration and increased female mating resistance (marked blue), trait values reduced, shortened or less successful compared to wild mating (marked brown), unchanged characters (yellow), and those not applicable or not visible faded out. The implications for sexual selection by either cryptic female choice (CFC) or sexually antagonistic coevolution (SAC) are given; CFC = The observed changes support sexual selection on titillators by cryptic female choice, SAC = The observed changes support sexual selection on titillators by sexually antagonistic coevolution, CFC/SAC: The observed changes are compatible with both cryptic female choice and sexually antagonistic coevolution.

### 3.1 Copula durations and titillator movements

**Figure 3:**
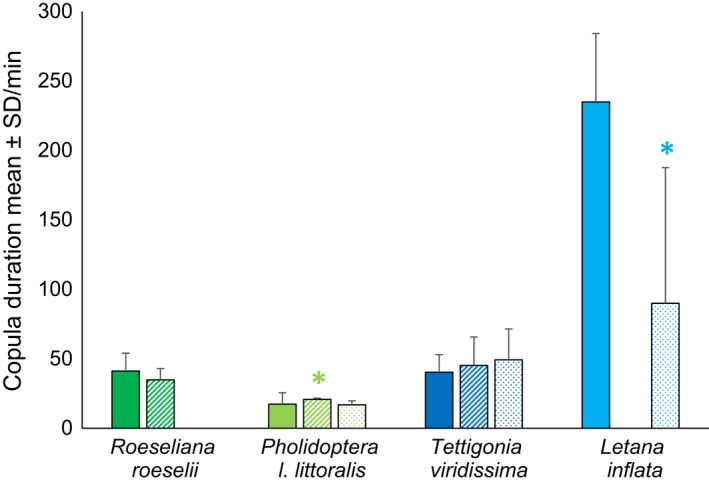
Comparison of copula durations (means ± *SD*) of unimpaired wild‐type (filled, left bars), asymmetrical (_p_T_‐1_ and _p_T_‐left spines_: striped, middle bars), and symmetrical manipulated males (_p_T_‐2_, _s_T_glued_: stippled, right bars) across the three Tettigoniinae species *Roeseliana roeselii* (*n* = 20), *Pholidoptera l. littoralis* (*n* = 13), *Tettigonia viridissima* (*n* = 20), and the Phaneropterinae *Letana inflata* (*n* = 16). * = significantly altered copula durations in manipulation experiments compared to wild types, for statistics see Table 6

**Figure 4:**
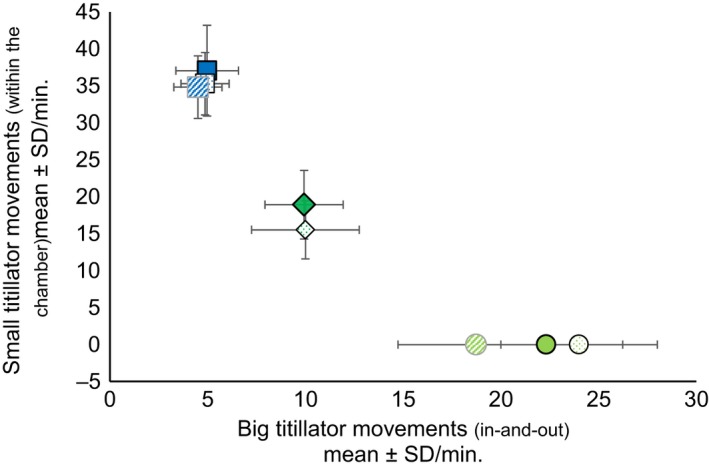
Mean number of copula movements (±*SD*) per minute for unmanipulated wild‐type (filled), asymmetrical (_p_T_‐1_ and _p_T_‐left spines_: striped), and symmetrical manipulated (_p_T_‐2_: stippled) males of the three Tettigoniinae species with paired titillators *Tettigonia viridissima* (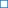), *Roeseliana roeselii* (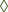), and *Pholidoptera l. littoralis* (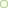). Males performed two different types of copulatory movements: big titillator movements with inserting and retracting titillators from the female genital chamber (“in‐and‐out”) and small titillator movements within the female's genital chamber

Copula durations (Figure 3) and the number of titillator movements (Figure 4) varied greatly between the four bushcricket species, but less so between wild‐type and manipulated matings (Tables 6 and 7).

*Roeseliana roeselii* wild‐type males exhibited a broad span of copula durations, ranging from 25.93 to 73.50 min (mean ± *SD*: 41.16 ± 12.87, *n* = 20) (Figure 3). During copulation, they moved their titillators 9.93 ± 2.00 times per minute (mean ± *SD*, *n* = 20) in‐and‐out of the female genital chamber and performed small movements within the female genital chamber at the double rate (18.92 ± 4.65 per minute, mean ± *SD*, *n* = 17) (Figure 4, Video S1). Copula duration was unaltered by titillator manipulations, whereas the number of titillator movements was reduced in symmetric males (_p_T_‐2_) by around 10 percent, but not in asymmetric males (Table 7, see statistics Tables 6).

Males of *Ph. l. littoralis* showed the shortest copulation duration of the three Tettigoniinae species, and wild‐type matings lasted 17.41 ± 8.17 min (mean ± *SD*, *n* = 13), which was less than half of the duration compared to the other Tettigoniinae species (Figure 3). Despite the short time, *Ph. l. littoralis* males inserted and retracted their titillators more often from the female genital chamber than males of the other species (Figure 4), with a frequency of 22.32 ± 3.92 movements per minute (mean ± *SD*, *n* = 12). This high rate in large titillator movements seems to be compensated by the total lack of small titillator movements within the female's genital chamber (Figure 4). Copula duration was increased by 20 percent for asymmetrically manipulated *Ph. l. littoralis* (_p_T_‐1_) males (see Table 6 for statistics), whereas titillator movements did not change (Tables 6 and 7).

*Tettigonia viridissima* had a similar copula duration as *R. roeselii*: The males needed between 23.35 and 64.73 min (mean ± *SD*: 40.39 ± 12.56, *n* = 20). During mating, males showed the lowest rate of in‐and‐out titillator movements of all our species (mean ± *SD*: 4.97 ± 1.60 times per minute, *n* = 20). In contrast to *Ph. l. littoralis*, the low number of larger (in‐and‐out) titillator movements was compensated for by the highest rate of small movements within the female genital chamber (37.05 ± 6.12 per minute; mean ± *SD*, *n* = 21). The three Tettigoniinae species therefore demonstrate a negative correlation between the number of big titillator movements in‐and‐out of the female genital chamber and the number of small rhythmic titillator movements inside the chamber (Figure 4).

The Phaneropterinae *L. inflata* showed extended copula (Figure 3), which lasted around four hours in wild‐type matings (mean ± *SD*: 234.90 ± 49.24 min, *n* = 16) before females were released. The copula duration was drastically reduced to 1.5 hr when mating with manipulated males (mean ± *SD*: 89.93 ± 97.72, *n* = 18; Table 6). Unfortunately, movements of the single titillator were not observable as male and female genitalia were tightly coupled while males used their modified cerci and subgenital plate to securely hold the females.

### 3.2 Female mating resistance and spermatophore transfer success

No female resistance was observed for females of *Ph. l. littoralis* or *T. viridissima* regardless of whether the males had asymmetric (_p_T_‐1_) or symmetric (_p_T_‐2_) alterations. In contrast, females of *R. roeselii* and *L. inflata* resisted mating attempts by titillator‐manipulated males (males of the former have paired titillators, while the latter has a single titillator).

**Figure 5:**
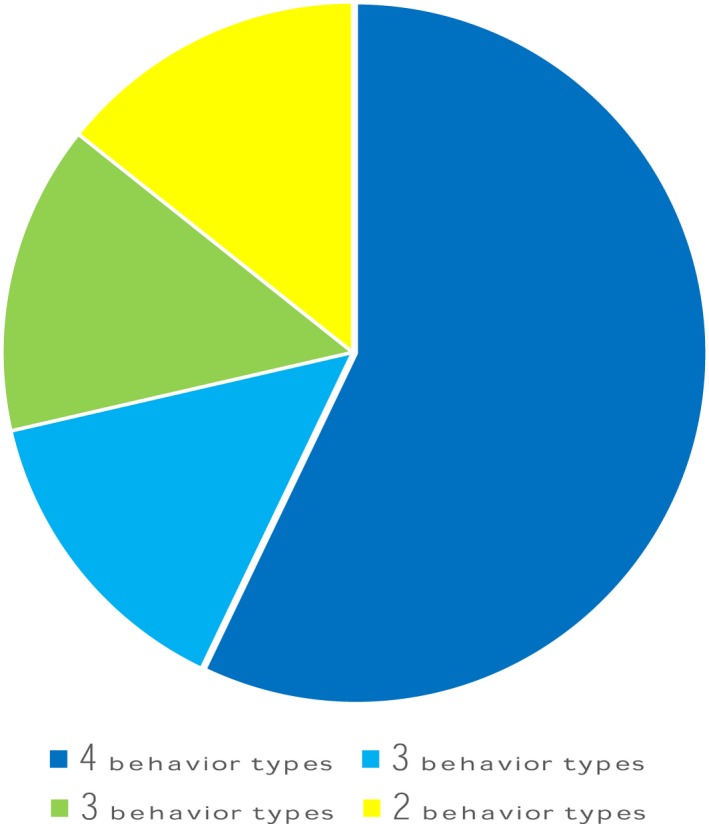
Resistance behavior of females of the Tettigoniinae species *Roeseliana roeselii*. One third of females mated with manipulated males (T_‐left spines_) showed resistance behavior by walking, jumping, kicking, or biting. Among the females that showed resistance, most females showed all four behavioral types, followed by three types of walking + jumping+kicking or jumping + kicking + biting or just two behaviors of walking + jumping

One third of *R. roeselii* females mated with manipulated males that had the spines of one titillator removed (T_‐left spines_) showed resistance behavior by walking, jumping, kicking, or biting prior to spermatophore transfer (Table 6: Fisher's exact test for the proportion of female resistance behaviors in manipulated vs. wild‐type matings: *p* = .0086, *n* = 41). These seven out of 21 females showed these resistance behaviors in different combinations (Figure 5), with the majority (57.1%) exhibiting all four behaviors (walking + jumping + kicking + biting the male). The remaining females showed two combinations of three (walking + jumping + kicking, 14.3%, jumping + kicking + biting the male 14.3%), or just the two behaviors of walking and jumping (14.3%). Such female resistance behavior resulted in separation of the couples in four cases. Three of the four couples reengaged in mating afterward. One female did not accept the males' attempts to reengage in copulation, and two females separated for a second time and did not attempt to mate further with the male. Three couples finished the mating attempt without spermatophore transfer, but this number was not significantly lower than in the wild‐type group (Fisher's exact test of pairs successfully finishing spermatophore transfer in females mated to manipulated (_p_T_‐left spines_) vs. wild‐type males: *p* = .23, *n* = 41). However, spermatophore transfer success gradually decreased with the amount of titillator manipulation, slightly, nonsignificantly reduced in asymmetric (_p_T_‐1_, _p_T_‐left spines_) but significantly reduced in symmetrically manipulated (_p_T_‐2_) males in previous experiments (Tables 6 and 7).

**Figure 6:**
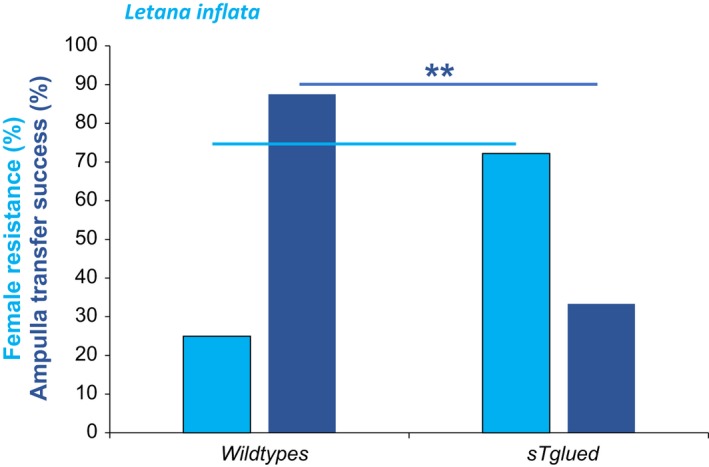
Female resistance behavior by walking (light blue) and ampulla transfer success (dark blue) in *Letana inflata* during copulation with wild‐type (*n* = 16) and with titillator‐glued males (*n* = 18). (Pearson chi‐square test: **p < .01)

In the single titillator‐bearing *L. inflata*, two thirds of the females mated with manipulated males (spines on the titillator covered with glue) walked during copula, a behavior only rarely shown by females mated with wild‐type males (Figure 6; Pearson chi‐square test: *χ* ^2^_1,34_ = 7.56, *p* = .0060). Moreover, this female resistance occurred earlier in the manipulated group (within the first 25.94 ± 54.26 min; mean ± *SD*, *n* = 13), but significantly later in females mated to wild‐type males (189.67 ± 41.48 min; mean ± *SD*, *n* = 4; Mann–Whitney *U* test: *U* = 1.0, *p* = .009, *n* = 13). Females also walked for extended periods when paired with modified males (Mann–Whitney *U* test: *U* = 5.50, *p* = .018, *n* = 13). Such disturbances during copula with titillator‐glued males led to shorter copula durations (*t* test: T_welch 25.75_ = 5.55, *p* < .001) and reduced ampulla transfer durations (*t* test: T_welch 13_ = 2.48, *p* = .028). In consequence, mating with titillator modified males increased the number of failed ampulla transfers in *L. inflata* (Pearson chi‐square test: *χ* ^2^_1,34_ = 10.26, *p* = .0014) and reduced the ampulla transfer success from nearly 90 to around 30 percent (Figure 6).

### 3.3 Titillator anchoring success

**Figure 7:**
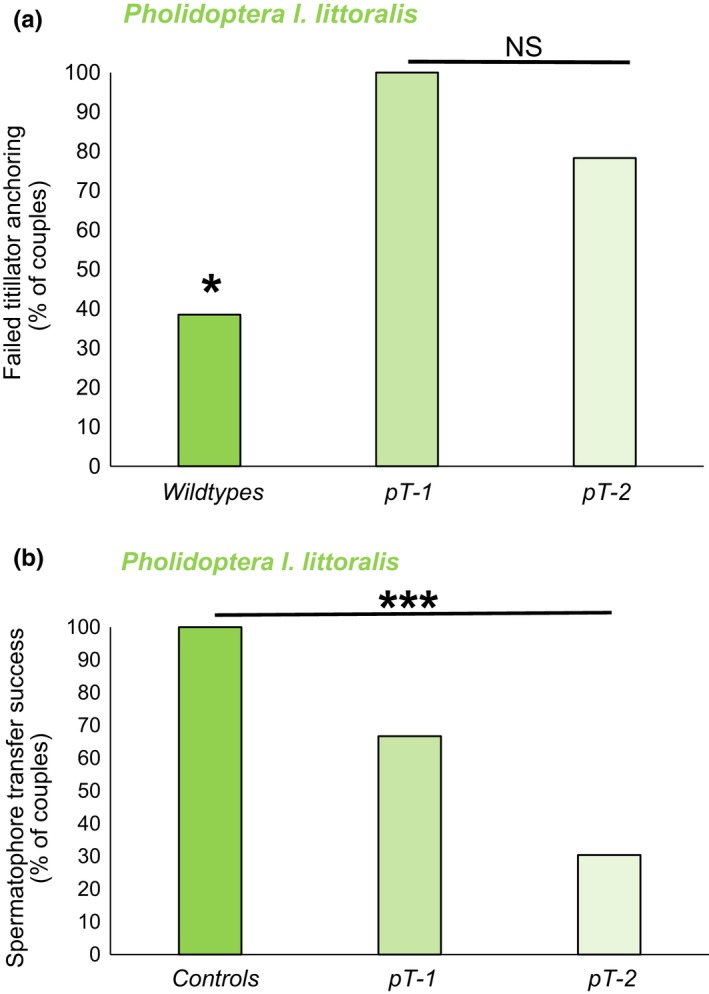
(a) Titillator anchoring and (b) success of spermatophore transfer for unmanipulated wild‐type males of *Pholidoptera l. littoralis* (*n* = 13) in comparison with one‐sided (_p_T_‐1_, *n* = 6) or both‐sided manipulated males (_p_T_‐2,_ *n* = 23). (Fisher's exact test: *p < .05, ***p < .001)

Shortening of the titillators in *Ph. l. littoralis* resulted in significant problems for males anchoring their titillators (Figure 7a); most males with shortened titillators were not able to copulate for long period without slipping out of the mating position (Fisher's exact test: *χ* ^2^ = 8.41, *p* = .011). This slipping out occurred both in males with one or two shortened titillators (post hoc test: wild type vs. _p_T_‐1_: *χ* ^2^_1,19_ = 6.38, *p* = .018; wild type vs. _p_T_‐2_: *χ* ^2^_1,36_ = 5.70, *p* = .030) and did not differ between the one‐ or both‐sided titillator modifications (_p_T_‐1_ vs. _p_T_‐2_: *p* = .55, *n* = 29). Furthermore, the copula duration was altered in titillator‐manipulated males (Kruskal–Wallis test *χ* ^2^_2,22_ = 6.79, *p* = .034). However, pair‐wise comparisons revealed that there was only a significant difference where males only had one titillator shortened. Copulation was longer for one‐sided modified (_p_T_‐1_) compared to wild‐type males (Dunn–Bonferroni post hoc test: wild type vs. T_‐1_: *z* = −2.59, *p* = .029), but did not change in both‐sided manipulated (_p_T_‐2_) males (wild type vs. _p_T_‐2_: *z* = −0.50, *p* > .05).

The inability to hold the mating position resulted in a failure of spermatophore transfer. In the manipulated groups, the success of the spermatophore transfer was reduced (Fisher's exact test: *χ* ^2^_2,42_ = 18.33, *p* < .001), especially for males with both titillators shortened (post hoc test: wild type vs. _p_T_‐2_: *χ* ^2^_1,36_ = 16.28, *p* < .001) (Figure 7b). This seems to be the consequence of a malfunction of manipulated titillators. Insertion of the male's titillators of *Pholidoptera l. littoralis *wild‐type males resulted in contact between male and female genitalia, while the retraction of the titillators was followed by a slow slipping out. As the next titillator insertion followed quickly (the wild‐type males inserted their titillators in mean 22 times per minute; Table 6, Figure 4), the couples in the wild‐type group separated seldom.

### 3.4 Postcopulatory behavior and outcomes

All observed changes due to titillator manipulations across the four species were restricted to the copulation phase. Postcopulatory female behaviors, such as the ingestion duration of the spermatophore or the ampulla, the female refractory period until the next mating or the number of eggs laid during this refractory period, remained unchanged (Tables 6 and 7).

## 4 DISCUSSION

Genitalia clearly evolved in response to sexual selection (Arnqvist, 1998; Eberhard, 1985, 1996; Hosken & Stockley, 2004; Rice, 1996). However, debate about the cause(s) of the outstanding evolutionary diversity of genitalia continues (Brennan, 2016; Brennan & Prum, 2015; Cordero & Eberhard, 2003; Eberhard, 2010a, 2010b, 2011; Joly & Schmitt, 2010; Simmons, 2014). Several competing hypotheses have been formulated. These include the lock‐and‐key hypothesis, which does not apply in most cases (Eberhard, 1985; Shapiro & Porter, 1989) and has only support in a very limited number of cases (Langerhans, Anderson, & Heinen‐Kay, 2016; Simmons, 2014), sperm competition (Parker, 1970; Simmons, 2001), cryptic female choice (CFC) (Arnqvist, 2014; Eberhard, 1996, 2010a, 2010b; Eberhard & Lehmann, 2019; Thornhill, 1983; Vahed, 2015), and sexually antagonistic coevolution (SAC) (Arnqvist & Rowe, 2005; Rice, 1996). The latter three hypotheses are not mutually exclusive, and the differences between them may be even less strict than they appear (Schilthuizen, 2003, 2013). Evidence is accumulating that the great complexity of animal genitalia is a result of not only different parts of the genitalia having different functions, but also being under different forms of selection (Kelly & Moore, 2016; Schilthuizen, 2003).

Our cross‐species comparison of four bushcricket species supports such a broadened view on evolutionary forces shaping insect genitalia, as mating‐related responses to titillator manipulations are species‐specific. In *R. roeselii*, the titillators apparently function as stimulators (Wulff et al., 2015, 2017), which are sensed by female receptors inside the female genital chamber (Wulff et al., 2018) and promote female acceptance of copulation and sperm transfer (Wulff et al. 2016; Wulff et al., 2018). Females' resistance behavior against males with asymmetrical spines in our new experiment is nearly identical to previous mating outcomes when males have one titillator removed (Wulff & Lehmann, 2016; Wulff et al., 2018). The symmetrical stimulation with the spines of both titillators seems to be crucial for determining whether females remain motionless with their genital folds open or disturb the copulation and try to prevent spermatophore transfer (Wulff & Lehmann, 2016; Wulff et al., 2018). The lack of symmetrical stimulation may therefore cause female rejection behavior. These results support our previous supposition that titillators in *R. roeselii* function as copulatory courtship devices (Wulff et al., 2015, 2018). The best explanation for these cumulative results seems to be female cryptic choice during copulation based on adequate stimulation (Eberhard, 1996; Eberhard & Lehmann, 2019). Furthermore, intact titillators seem to have an additional mechanical function, namely to support the spermatophore transfer, as spermatophore transfer success was lower for males who had both of their titillators altered (Wulff & Lehmann, 2016). This indicates an additional influence of sexually antagonistic coevolution on the *R. roeselii* titillators.

While the paired titillators of the other two Tettigoniinae *Ph. l. littoralis* and *T. viridissima* are morphologically similar to those of *R. roeselii* (Lehmann et al., 2017; Vahed et al., 2011; Figure 1), they do not appear to play the same role. This might suggest that in this species titillators either do not act as stimulators or alternatively that they have effects that do not impact on mating success. So titillator movements seem to be species‐specifically sensed by the females and trigger different processes. In *T. viridissima*, neither symmetrical nor asymmetrical titillator alterations substantially affected female behavioral responses as none of our measured parameters during and after the mating are altered. Consequently, the importance of titillators for mating in this species is unclear. However, as the titillators and the surrounding phallobasis are moved in concert with a fast rhythm, the movements of the phallobasis alone might be sufficient to stimulate the females. Therefore, the possibility of cryptic female choice cannot be excluded. It is clear from our results that we need deeper insights into the mating system of this species to understand the titillator function. The challenge is that finding an effect is easy to interpret, but the lack of a female response does not exclude the possibility that copulatory or postcopulatory selection exists (Eberhard, 2011).

The third Tettigoniinae, *Ph. l. littoralis*, uses titillators as mechanical anchors. Each titillator insertion induces an approach of the genitalia, while the retraction results in a slow slipping out of the genital chamber. This slow separation movement is counteracted by rapid titillator reinsertion, resulting in a high titillator movement frequency. In the wild‐type mating experiments, titillator movement only occasionally leads to a separation of the copulating pair. As the females allow them to remount, all wild‐type males transfer their spermatophore. In contrast, experimental shortening of the titillators results in males slipping out regularly, regardless of whether one or both titillators are altered. Males could keep the mating position only for short periods, and several mating partners separate without being able to transfer the spermatophore. As a result, spermatophore transfer is reduced. The effect is only significant when both titillators are shortened. We therefore conclude that the titillators in *Ph. l. littoralis* have a function as anchors, mechanically facilitating male attachment, while also assisting spermatophore transfer. Such genitalia anchoring is reported for several insect species (Simmons, 2014) and might be selected for by sexually antagonistic coevolution (SAC). Interestingly, titillator anchoring is found only in one of the four bushcricket species tested by us. However, an anchoring function might not explain the repeated retraction and reinsertion of the titillators. The quick in‐and‐out movement of the titillators therefore hints to some stimulating function as well, even if we have not identified the triggered female copulatory or postcopulatory responses yet.

In our out‐group species from the subfamily Phaneropterinae, *L. inflata*, nonconsensual mating is possible, where males grasp the female on the ventral part of the abdomen and then slowly move downwards until reaching mating position (Heller & Liu, 2015). Females who move while the male is grasping her abdomen can be injured by the spines on the male's cerci (we observed two out of seven females who struggled during the grasping stage were bleeding afterward). Female resistance at this point therefore can be risky. In contrast to most bushcrickets species, the mating partners do not separate immediately after ampulla transfer but stay in a lengthy copula until the sperm have entered the female spermatheca (Lehmann et al., 2016). In this respect, *L. inflata* is similar to several other bushcricket species who have replaced the sperm‐protecting function of the costly spermatophylax (Lehmann, 2012; Lehmann et al., 2018) with prolonged postcopulatory mate guarding (Vahed, Gilbert, Weissman, & Barrientos‐Lozano, 2014). However, in our experiments a significant number of females resisted manipulated males, resulting in reduced copula duration. Therefore, *L. inflata* also demonstrates cryptic female choice, as properly stimulated females refrain resistance and accept a proper attachment of the sperm‐containing ampulla.

Comparing the four species demonstrates that titillator function and the reactions toward titillator‐manipulated males show no unifying pattern. Manipulation of the male's titillators had diverse effects. These include affecting female stimulation, the suppression of female resistance to allow stable male fixation, and mechanical support of spermatophore attachment. It is useful to study genital behavior across species in a robust phylogenetic framework, but in contrast to the general expectation of shared outcomes in more closely related species (Eberhard, 2011), our results are independent of the phylogenetic relationships (Hawlitschek et al., 2017; Mugleston et al., 2018). As no clear relationship between the titillator morphology and the responses toward their alterations was found, closer study of both sexes genitalia function for each species is warranted. This is a challenge, as most research focuses on genitalia morphology in males (reviewed in Simmons, 2014) and females as well (Sloan & Simmons, 2019), despite the consideration by Eberhard (2011) that sexual selection on genitalia might act on different female responses. Studying the function of genitalia therefore needs a better understanding of their action. Advanced imaging technologies exist that allow to study the hidden nature of genitalia action inside the female. For example, we have applied the snap‐frozen technique in combination with static µCT (Wulff et al., 2015) and synchrotron‐assisted live scans of the internal mechanisms in our model species *R. roeselii* (2017). Applying these advanced imaging techniques successfully revealed the internal mechanisms and made the otherwise hidden genital movements of titillators visible. As understanding the function is crucial to develop testable behavioral paradigms, we strongly encourage researchers of genitalia to move beyond describing static morphologies, which unfortunately still prevails as the major information published for most insect and arthropod species.

After studying genital functions, the next necessary step is to test behaviors of the mating partners. The notion that mating behaviors cannot be deduced from morphology alone, but have diversified independently from morphology (Eberhard, 2011), is well supported by our data; despite morphological similarity between the three Tettigoniinae species belonging to the same titillator morphotype (Lehmann et al., 2017; Vahed et al., 2011), the behavioral alterations associated with titillator manipulations vary largely. Such plasticity in behavioral responses despite morphological similarities can be attributed to the filter function of the nervous system, showing that behavior connects evolutionary selection pressures with individuals' performance (Orr & Garland, 2017). Again, it is less surprising that genital behavioral parameters and responses vary between species. Similar results have been observed for five *Glossina* fly species (Briceño & Eberhard, 2009a, 2009b; Briceño & Eberhard, 2017; Briceño, Eberhard, Chinea‐Cano, Wegrzynek, & Santos Rolo, 2016). The copula duration of our bushcrickets is highly species‐specific, varying from moderately short in *Ph. l. littoralis* to very long in *L. inflata.* In matings involving males with altered titillators, the copula duration is shortened in the long copulations of *L. inflata*, but prolonged in the short copulations of *Ph. l. littoralis*. Whether this response is a general pattern reflecting female cryptic choice selecting against males bearing unfavorable titillators might be analyzed across a greater number of species. A second behavioral response is found in *L. inflata* for the ampulla transfer duration, which, in accordance with the shorter copula duration, is also reduced in matings with titillator‐manipulated males. Despite any sexual selection implications, the combined number of small and large titillator movements seems to be constrained; this is reflected in a negative correlation between the number of large versus the number of small titillator movements across species. It can be assumed that the physical capability for movements limits the combined number of small and large titillator movements.

It is possible that titillator movements are a character representing male fitness, which would make the titillator capacity an honest male signal detectable by females. In this case, the female responses of both *L. inflata* and *R. roeselii* can be attributed to cryptic female choice, as females resist males with altered titillators, reducing the sperm transfer success. The exhibited range of female rejection behaviors is plastic and includes female moving during copulation, biting, and a range of other behaviors.

In conclusion, it might help to widen our theoretical approaches and analyze the interplay between males and females during mating within a communication framework, as mating includes the production, hence exchange, and detection by the nervous system, hence reception, of copulatory signals (Briceño & Eberhard, 2017; Rodriguez, 2015). The bushcricket titillators might be a good example for such an approach, as the evidence for the four tested species suggests the evolution of genitalia under a sexual selection mosaic of mainly cryptic female choice, some evidence as well as for sexually antagonistic coevolution, or even a mosaic of both acting within the same species.

## CONFLICT OF INTEREST

None declared.

## AUTHOR CONTRIBUTIONS

NW and GL jointly designed the study. NW collected and reared the specimens, performed the experiments, and initially analyzed the data. GL supervised the study. Both NW and GL interpreted the results, wrote the manuscript, and approved the final version of the manuscript before submission.

## Data Availability

The behavioral and body mass data from the manuscript are archived with Dryad (https://doi.org/10.5061/dryad.crjdfn31f). Sampling locations are included in Table 1 of the Section 2.
