# Supplementary material for: Sexual selection on bushcricket genitalia operates in a mosaic pattern
Source: Ecol Evol. 2020 Feb 25;10(5):2320–38. doi: 10.1002/ece3.6025 (PMC7069301; doi:10.1002/ece3.6025)
Supplement: Supplementary file 2 [file ECE3-10-2320-s002.docx]

**Video 1**: Unrestrained mating in the bushcricket *R. roeselii* with rhythmic titillator movements alternating between in-and-out (big) and within (small) the female’s genital chamber.
